# SARS-CoV-2 hijacks macropinocytosis to facilitate its entry and promote viral spike–mediated cell-to-cell fusion

**DOI:** 10.1016/j.jbc.2022.102511

**Published:** 2022-09-19

**Authors:** Yu-Yuan Zhang, Ronghui Liang, Shu-Jie Wang, Zi-Wei Ye, Tong-Yun Wang, Meng Chen, Jianbo Liu, Lei Na, Yue-Lin Yang, Yong-Bo Yang, Shuofeng Yuan, Xin Yin, Xue-Hui Cai, Yan-Dong Tang

**Affiliations:** 1 State Key Laboratory of Veterinary Biotechnology, Harbin Veterinary Research Institute of Chinese Academy of Agricultural Sciences, Harbin, China; 2 Department of Microbiology, Li Ka Shing Faculty of Medicine, The University of Hong Kong, Pokfulam, Hong Kong SAR, China; 3 State Key Laboratory of Emerging Infectious Diseases, Li Ka Shing Faculty of Medicine, The University of Hong Kong, Pokfulam, Hong Kong SAR, China

**Keywords:** SARS-CoV-2, macropinocytosis, entry, cell-to-cell, fusion, ACE2, angiotensin-converting enzyme 2, CCK-8, Cell Counting Kit-8, COVID-19, coronavirus disease 2019, CPZ, chlorpromazine hydrochloride, Cyto-B, cytochalasin B, DMSO, dimethyl sulfoxide, EBOV, ebolavirus, EGFP, enhanced GFP, EGFR, epidermal growth factor receptor, EIPA, 5-(*N*-ethyl-*N*-isopropyl) amiloride, GP, glycoprotein, HEK293T, human embryonic kidney 293T cell line, hpi, hours postinfection, hpt, hours post-transfection, MβCD, methyl-β-cyclodextrin, MHV, murine hepatitis virus, MOI, multiplicity of infection, NHE, Na^+^/H^+^ exchange, Pak, p21-activated kinase, qRT–PCR, quantitative RT‒PCR, SARS-CoV-2, severe acute respiratory syndrome coronavirus 2, TMPRSS2, transmembrane serine protease 2, TMR, tetramethylrhodamine, VSV, vesicular stomatitis virus

## Abstract

Revealing the mechanisms of severe acute respiratory syndrome coronavirus 2 (SARS-CoV-2) entry and cell-to-cell spread might provide insights for understanding the underlying mechanisms of viral pathogenesis, tropism, and virulence. The signaling pathways involved in SARS-CoV-2 entry and viral spike–mediated cell-to-cell fusion remain elusive. In the current study, we found that macropinocytosis inhibitors significantly suppressed SARS-CoV-2 infection at both the entry and viral spike–mediated cell-to-cell fusion steps. We demonstrated that SARS-CoV-2 entry required the small GTPase Rac1 and its effector kinase p21-activated kinase 1 by dominant-negative and RNAi assays in human embryonic kidney 293T–angiotensin-converting enzyme 2 cells and that the serine protease transmembrane serine protease 2 reversed the decrease in SARS-CoV-2 entry caused by the macropinocytosis inhibitors. Moreover, in the cell-to-cell fusion assay, we confirmed that macropinocytosis inhibitors significantly decreased viral spike–mediated cell-to-cell fusion. Overall, we provided evidence that SARS-CoV-2 utilizes a macropinocytosis pathway to enter target cells and to efficiently promote viral spike–mediated cell-to-cell fusion.

Severe acute respiratory syndrome coronavirus 2 (SARS-CoV-2) poses huge public health threats worldwide and is still at pandemic levels in most countries (1, 2). Cellular entry and spread from cell-to-cell are key steps in the SARS-CoV-2 life cycle and pivotally determine viral infectivity and pathogenesis *in vivo* (3, 4, 5). Angiotensin-converting enzyme 2 (ACE2) was identified as the primary cell entry receptor for SARS-CoV-1 and SARS-CoV-2 (1, 6, 7, 8). The cell entry mechanisms of SARS-CoV-2 have also been extensively explored (7, 9). SARS-CoV-2 entry is believed to be initiated by the S1 domain within the S protein through binding to ACE2, followed by independent entry pathways: direct fusion between the viral membrane and plasma membrane at the cell surface and endocytosis that relies on endosomal-dependent uptake and final fusion between viral and lysosomal membranes in lysosomes. Both entry pathways require S protein priming, which is mediated by either the lysosomal protease cathepsin for the endocytosis pathway (7, 9) or cellular serine proteases, such as transmembrane serine protease 2 (TMPRSS2), for the direct fusion of viral and plasma membranes (7, 10). For the endocytosis pathway, cathepsin L in lysosomes may be the predominant lysosomal protease required for SARS-CoV-2 S protein priming (11).

Endocytosis can be classified into several types: clathrin-mediated endocytosis, caveolae-dependent endocytosis, phagocytosis, lipid raft–mediated endocytosis, and macropinocytosis (12, 13). Various viruses may take advantage of these endocytosis pathways for viral entry in a cell type–dependent manner (14). Macropinocytosis is the actin-dependent endocytic process responsible for nonspecific uptake of fluid, solutes, membranes, ligands, and smaller particles (including viruses) attached to the plasma membrane (12, 15). Macropinocytosis-mediated viral entry requires virus binding, leading to intracellular signaling activation. The activated signaling further induces plasma membrane protrusion, vacuole closure, and vacuole formation. Finally, macropinosomes are formed and trafficked to lysosomes (15). In this process, Rac1-GTPase activation plays an important role in macropinocytosis: Rac1 regulates macropinocytosis by interacting with its specific effectors, the p21-activated kinases (Paks), thus modulating actin cytoskeleton dynamics, which finally trigger membrane ruffling in the cell (15). Pak1 is a serine/threonine kinase that can be activated by Rac1 or Cdc42. Na^+^/H^+^ exchange (NHE) activity is required to achieve a necessary H^+^ concentration transiently in the vicinity of the membrane to stimulate cytoskeleton remodeling (15).

Many viruses, including HIV-1, herpes simplex virus 1, Kaposi's sarcoma–associated herpesvirus, vaccinia virus, species B human adenovirus serotype 3, echovirus 1, and group B Coxsackieviruses, have been reported to utilize macropinocytosis to enter host cells (12, 14, 16, 17).

Interestingly, for several coronaviruses, such as murine hepatitis virus (MHV) and SARS-CoV-1, macropinocytosis was found to mainly facilitate CoV infection through enhanced cell-to-cell spreading rather than by promoting virus entry (18). Recent studies illustrated that clathrin-mediated endocytosis was involved in SARS-CoV-2 entry (19); however, whether SARS-CoV-2 utilizes macropinocytosis to enter host cells has not been fully investigated (20, 21).

SARS-CoV-2 spike–mediated cell-to-cell fusion may be very important for its pathogenesis. In almost 90% of patients who died from coronavirus disease 2019 (COVID-19), atypical cells with syncytia showing a large cytoplasm containing a variable number of nuclei ranging from two to more than 20 were observed (22). Viral spike–mediated cell-to-cell fusion may facilitate viral spread and evasion of inhibition by neutralizing antibodies (23, 24). Potential drugs targeting viral spike–mediated cell-to-cell fusion may become an alternative therapy for COVID-19 (22).

In this study, we systemically evaluated the roles of macropinocytosis in SARS-CoV-2 entry and viral spike–mediated cell-to-cell fusion. Using SARS-CoV-2 spike pseudotyped virus and authentic virus infection models, we found that SARS-CoV-2 could hijack macropinocytosis to facilitate its entry. NHE, Rho GTPase Rac1, and its downstream Pak1 were essential for SARS-CoV-2 entry into human embryonic kidney 293T (HEK293T)–ACE2 cells. Furthermore, using a cell-to-cell fusion model, we also demonstrated that viral spike–mediated cell-to-cell fusion was regulated by macropinocytosis. This knowledge is likely to provide insight into understanding the life cycle of SARS-CoV-2 and may be helpful to develop potential therapeutic agents.

## Results

### Replication of SARS-CoV-2 was inhibited by a macropinocytosis inhibitor

**Figure 1:**
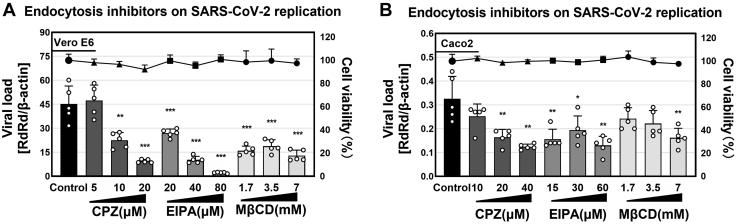
**Macropinocytosis is involved in the life cycle of SARS-CoV-2.***A*, Vero E6 cells were pretreated with EIPA, MβCD, and CPZ for 45 min and then infected with SARS-CoV-2 at an MOI of 0.05. Viral copies in cell lysates were measured by qPCR at 6 h postinfection (n = 5, mean ± SD). The cell viability of the indicated inhibitors was evaluated individually using a Cell Counting Kit 8 (CCK-8). *B*, pretreated Caco2 cells (EIPA, MβCD, and CPZ) were infected with SARS-CoV-2 at an MOI of 0.5, and viral copies were analyzed by qPCR. The cell viability of the indicated inhibitors was evaluated individually using a CCK-8. ns, nonsignificant; ∗*p* < 0.05; ∗∗*p* < 0.01; ∗∗∗*p* < 0.001. The results are the mean ± SD. CPZ, chlorpromazine hydrochloride; EIPA, 5-(*N*-ethyl-*N*-isopropyl) amiloride; MβCD, methyl-β-cyclodextrin; MOI, multiplicity of infection; qPCR, quantitative PCR; SARS-CoV-2, severe acute respiratory syndrome coronavirus 2.

To test whether macropinocytosis was involved in the SARS-CoV-2 life cycle, we used a panel of compounds to treat the cells prior to viral infection, which includes 5-(*N*-ethyl-*N*-isopropyl) amiloride (EIPA), an inhibitor of macropinocytosis that targets NHE to block the macropinocytosis pathway, chlorpromazine hydrochloride (CPZ), a commonly used compound to inhibit clathrin-mediated endocytosis, and methyl-β-cyclodextrin (MβCD), an inhibitor of cholesterol-rich lipid rafts that depletes cholesterol. The cell viability after treatment with these inhibitors was evaluated individually using a Cell Counting Kit-8 (CCK-8; Dojindo, catalog number CK04; Fig. 1, *A* and *B*). After treatment, Vero E6 cells were infected with SARS-CoV-2 at a multiplicity of infection (MOI) of 0.05. Viral copies in cell lysates were quantified by quantitative RT‒PCR (qRT–PCR). The results indicated that EIPA, MβCD, and CPZ significantly inhibited SARS-CoV-2 production compared with the dimethyl sulfoxide (DMSO) control (Fig. 1*A*). For EIPA and CPZ, the inhibition occurred in a dose-dependent manner, which indicated that both macropinocytosis and clathrin-mediated endocytosis were involved in SARS-CoV-2 replication in Vero E6 cells. However, all doses of MβCD we tested showed similar inhibition levels. We speculated that a small amount of MβCD may effectively deplete cholesterol in lipid rafts. Since TMPRSS2 can mediate the direct fusion of viral and plasma membranes in several cell lines (7, 10), we next tested whether the aforementioned tested pathways were involved in SARS-CoV-2 replication in TMPRSS2-expressing cells. Caco-2 cells have been demonstrated to express high levels of endogenous TMPRSS2 and have been widely used as a SARS-CoV-2 infection model (25, 26). We found that all these inhibitors could inhibit SARS-CoV-2 replication in Caco-2 cells (Fig. 1*B*). However, the inhibitory activity was less potent than that in Vero E6 cells, suggesting that TMPRSS2-mediated direct fusion of the viral and plasma membranes and endocytosis-mediated entry may contribute to SARS-CoV-2 entry into Caco-2 cells. Taken together, these results indicated that macropinocytosis, clathrin-mediated endocytosis, and cholesterol-rich lipid rafts were all involved in SARS-CoV-2 replication.

### Cell entry of SARS-CoV-2 was inhibited by a macropinocytosis inhibitor

**Figure 2:**
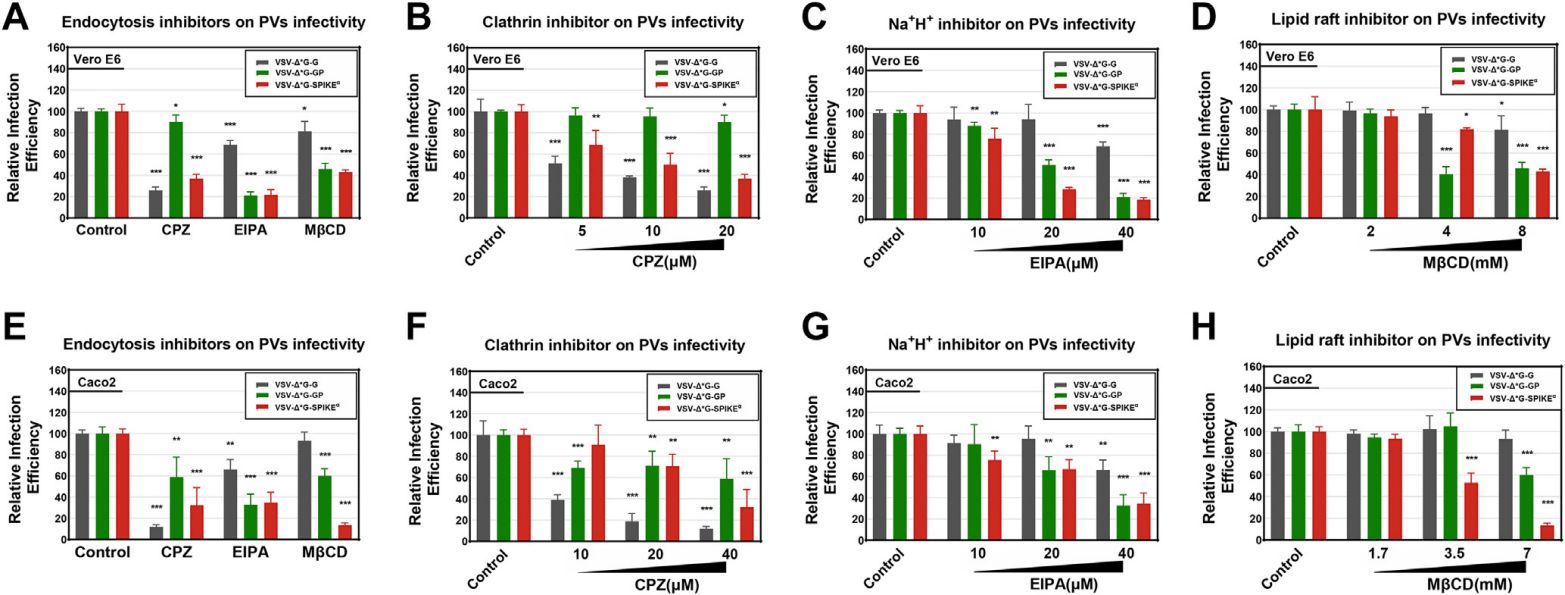
**EIPA significantly affects SARS-CoV-2 at the entry step.** Inhibitory effects of EIPA, MβCD, and CPZ on pseudovirus entry in (*A*) Vero E6 cells or (*E*) Caco2 cells. Cells pretreated with EIPA, MβCD, and CPZ were infected with VSV-ΔG-Luc pseudovirus for 80 min in the presence of inhibitors. DMSO-treated cells were used as a control. Twenty-four hours later, the cells were lysed, and virus infection was determined by measuring luciferase activity (n = 4, mean ± SD). *B*, Vero E6 cells or (*F*) Caco2 cells pretreated with the indicated concentrations of chlorpromazine (CPZ) were infected with the indicated pseudovirus. *C*, Vero E6 cells or (*G*) Caco2 cells pretreated with the indicated concentrations of EIPA were infected with the indicated pseudovirus. *D*, Vero E6 cells or (*H*) Caco2 cells pretreated with the indicated concentrations of MβCD were infected with the indicated pseudovirus. Luciferase values were normalized to the DMSO-treated control. ns, nonsignificant; ∗*p* < 0.05; ∗∗*p* < 0.01; and ∗∗∗*p* < 0.001. CPZ, chlorpromazine hydrochloride; DMSO, dimethyl sulfoxide; EIPA, 5-(*N*-ethyl-*N*-isopropyl) amiloride; MβCD, methyl-β-cyclodextrin; SARS-CoV-2, severe acute respiratory syndrome coronavirus 2; VSV, vesicular stomatitis virus.

To further investigate the role of macropinocytosis in SARS-CoV-2 entry, we synthesized the codon-optimized spike gene of SARS-CoV-2 and successfully produced a pseudotyped virus with a firefly luciferase reporter based on vesicular stomatitis virus (VSV)–pseudotyped SARS-CoV-2 (27). Ebolavirus (EBOV) has been reported to enter cells through macropinocytosis in a glycoprotein (GP)-dependent manner (28), and VSV enters cells in the clathrin-mediated endocytosis pathway (29). Therefore, in this study, we used EBOV-GP and VSV-G envelope pseudotyped viruses as positive and negative controls, respectively. Next, we evaluated whether EIPA influenced SARS-CoV-2 entry in Vero E6 cells, and CPZ and MβCD were used as controls. The results showed that EIPA significantly decreased EBOV-GP and SARS-CoV-2-spike pseudovirus entry (Fig. 2*A*), although the inhibition of VSV-G pseudovirus entry was approximately 25 to 30%. As expected, CPZ significantly inhibited VSV-G and SARS-CoV-2-spike pseudovirus entry, and the decrease in EBOV-GP pseudovirus entry was only approximately 10% (Fig. 2*A*). MβCD also potently decreased EBOV-GP and SARS-CoV-2-spike pseudovirus entry (Fig. 2*A*). We further confirmed these observations in Caco-2 cells (Fig. 2*E*). More importantly, these inhibitors worked in a dose-dependent manner (Fig. 2, *B*–*D* and *F*–*H*). Collectively, these results indicated that SARS-CoV-2 entry relied on macropinocytosis, clathrin-mediated endocytosis, and cholesterol-rich lipid rafts.

**Figure 3:**
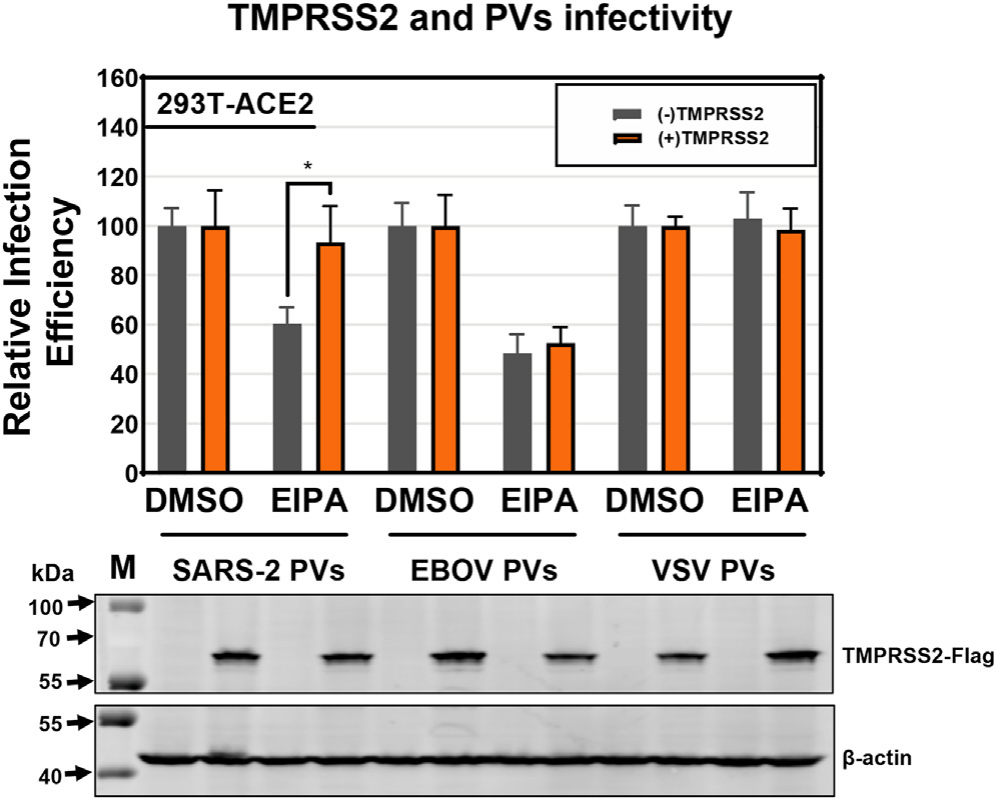
**Inhibitory effects of EIPA on pseudovirus entry are altered by TMPRSS2.** HEK293T–ACE2 cells were transfected with TMPRSS2 or the empty vector. After 24 h, the cells were treated with EIPA (100 μM) for 30 min and then infected with the indicated pseudovirus. Luciferase activity was measured at 24 hpi. All experiments were repeated at least three times (n = 4, mean ± SD). The entry of the control group was set as 100%. The expression of TMPRSS2 was verified by Western blotting. ns, nonsignificant; ∗*p* < 0.05; ∗∗*p* < 0.01; ∗∗∗*p* < 0.001. ACE2, angiotensin-converting enzyme 2; EIPA, 5-(*N*-ethyl-*N*-isopropyl) amiloride; HEK293T, human embryonic kidney 293T cell line; hpi, hours postinfection; TMPRSS2, transmembrane serine protease 2.

Next, we tested whether TMPRSS2 could restore the entry of SARS-CoV-2 pseudoviruses inhibited by EIPA. EBOV-GP and VSV-G pseudoviruses were used as controls, and HEK293T–ACE2 cells were used as the infection model. TMPRSS2 expression was confirmed by Western blotting (Fig. 3). We found that the inhibitory effect of EIPA on pseudovirus entry mediated by the SARS-CoV-2 spike protein was significantly restored by supplementation with TMPRSS2 (Fig. 3). As expected, TMPRSS2 had no effect on the entry of EBOV-GP and VSV-G pseudoviruses.

### Epidermal growth factor receptor and PI3K inhibitors blocked macropinocytosis-mediated SARS-CoV-2 entry

**Figure 4:**
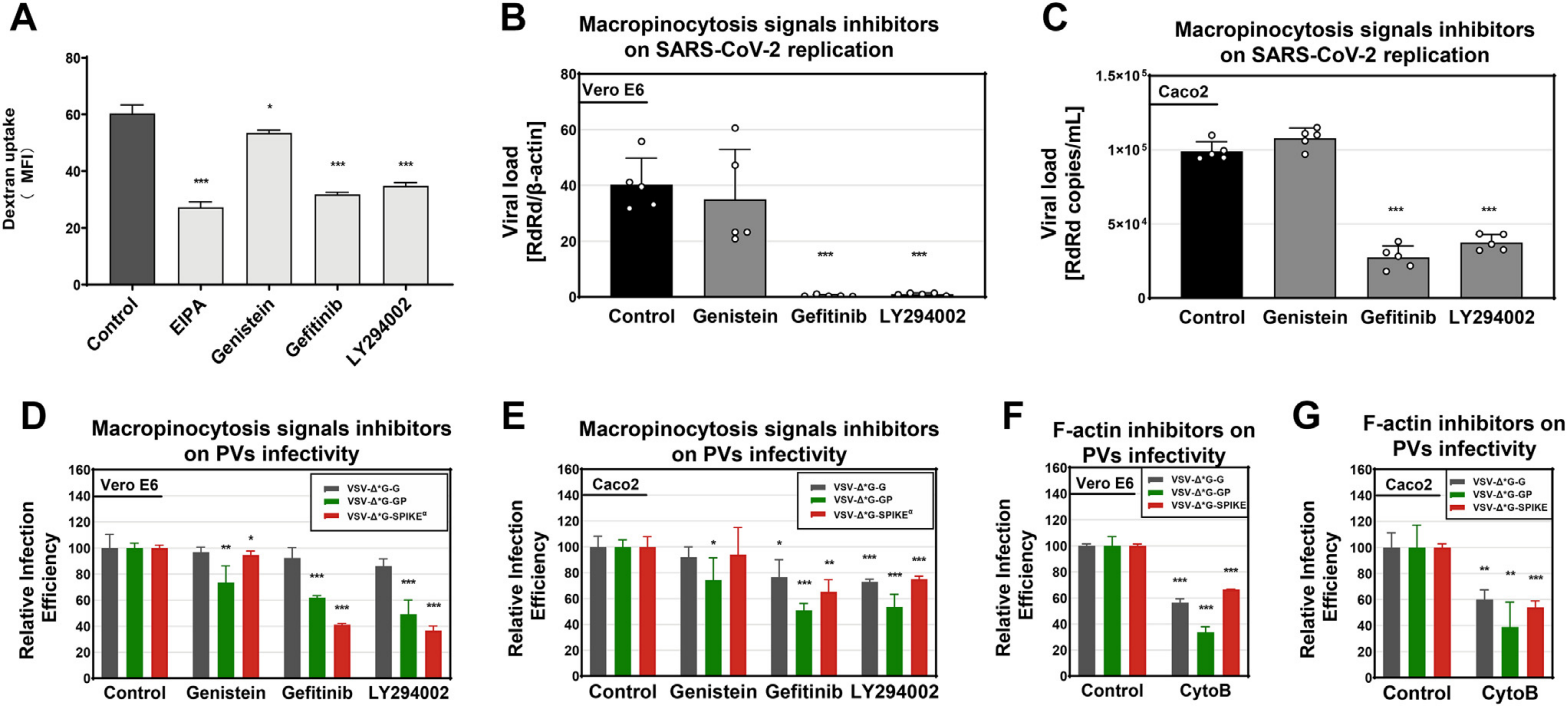
**EGFR and PI3K inhibitors block macropinocytosis-mediated SARS-CoV-2 entry.***A*, EGFR and PI3K inhibitors inhibited 70 kDa dextran uptake in Vero E6 cells. Vero E6 cells were treated with EIPA, genistein (an RTK inhibitor, 200 μM), gefitinib (an EGFR inhibitor, 40 μM), or LY294002 (a PI3K inhibitor, 80 μM). Control cells were treated with DMSO. The internalized fluorescent dextran was measured in ten individual cells by using a confocal laser scanning microscope. Each experiment was performed in triplicate, and the results are presented as the mean ± SD. *B*, Vero E6 cells were pretreated with genistein, gefitinib, and LY294002 for 30 min and then infected with SARS-CoV-2 at an MOI of 0.05. Viral copies in cell lysates were measured by qPCR. *C*, Caco2 cells pretreated with genistein (200 μM), gefitinib (40 μM), or LY294002 (80 μM) were infected with SARS-CoV-2 at an MOI of 0.5, and viral copies in the supernatants were measured by qPCR. *D*, pseudovirus entry in Vero E6 cells or (*E*) Caco2 cells pretreated with genistein, gefitinib, LY294002. Luciferase values were normalized to DMSO-treated control cells. *F*, pseudovirus entry in Vero E6 cells pretreated with cytochalasin B (1 μM). Luciferase values were normalized to control cells. *G*, pseudovirus entry in Caco2 cells pretreated with cytochalasin B (20 μM). Luciferase values were normalized to control cells. ns, nonsignificant; ∗*p* < 0.05; ∗∗*p* < 0.01; ∗∗∗*p* < 0.001. Error bars represent the SEM. DMSO, dimethyl sulfoxide; EGFR, epidermal growth factor receptor; MOI, multiplicity of infection; qPCR, quantitative PCR; RTK, receptor tyrosine inhibitor; SARS-CoV-2, severe acute respiratory syndrome coronavirus 2.

Macropinocytosis requires coordinated activation of multiple signaling pathways for initiation (12, 15). Signaling through epidermal growth factor receptor (EGFR) is essential for inducing macropinocytosis in several systems (18, 30). To test whether EGFR activation was required for SARS-CoV-2 entry, the EGFR inhibitor gefitinib was utilized to specifically inhibit EGFR autophosphorylation and prevent EGFR activation (31). PI3K has been implicated in several stages of macropinocytosis, from membrane protrusion to macropinosome trafficking and fusion (15). In addition, LY294002, a PI3K inhibitor that blocks macropinosome closure, was included for testing (32). We also evaluated a general tyrosine kinase inhibitor, genistein, for its effect on the entry of SARS-CoV-2. We first tested whether these inhibitors could effectively inhibit macropinocytosis. We used dextran MW 10,000 as a macropinocytosis marker. When macropinocytosis occurs, dextran is taken up by cells. We found that when cells were treated with EIPA, gefitinib, and LY294002, the uptake of dextran was effectively inhibited; conversely, genistein had less of an effect on the uptake of dextran (Fig. 4*A*). We next tested whether genistein, gefitinib, and LY294002 could inhibit SARS-CoV-2 replication. We found that gefitinib and LY294002 substantially reduced SARS-CoV-2 replication in Vero E6 and Caco-2 cells, whereas genistein had no effect on SARS-CoV-2 replication (Fig. 4, *B* and *C*). These results indicate that genistein may not be an ideal tyrosine kinase inhibitor for studying macropinocytosis, as has been demonstrated in vaccinia and Nipah viruses (15, 33, 34). We next evaluated whether these inhibitors played a role in SARS-CoV-2 entry. We found that gefitinib and LY294002 substantially reduced EBOV-GP and SARS-CoV-2-spike pseudovirus entry but not VSV-G pseudovirus entry in Vero E6 cells (Fig. 4*D*). We observed a similar phenomenon in Caco-2 cells (Fig. 4*E*). Since actin polymerization is a key step for macropinocytosis and cytochalasin B (Cyto-B) inhibits actin polymerization, which is required for macropinocytosis (12, 35, 36), we further investigated whether actin polymerization could affect SARS-CoV-2 entry. We found that Cyto-B could significantly inhibit EBOV-GP and SARS-CoV-2-spike pseudovirus entry in both Vero E6 and Caco-2 cells (Fig. 4, *F* and *G*). Interestingly, VSV-G pseudovirus entry was also inhibited by Cyto-B (Fig. 4, *F* and *G*), indicating that actin polymerization is essential for virus infection.

### Macropinocytosis-mediated SARS-CoV-2 entry was dependent on the small GTPase Rac1 and Pak1 kinases

**Figure 5:**
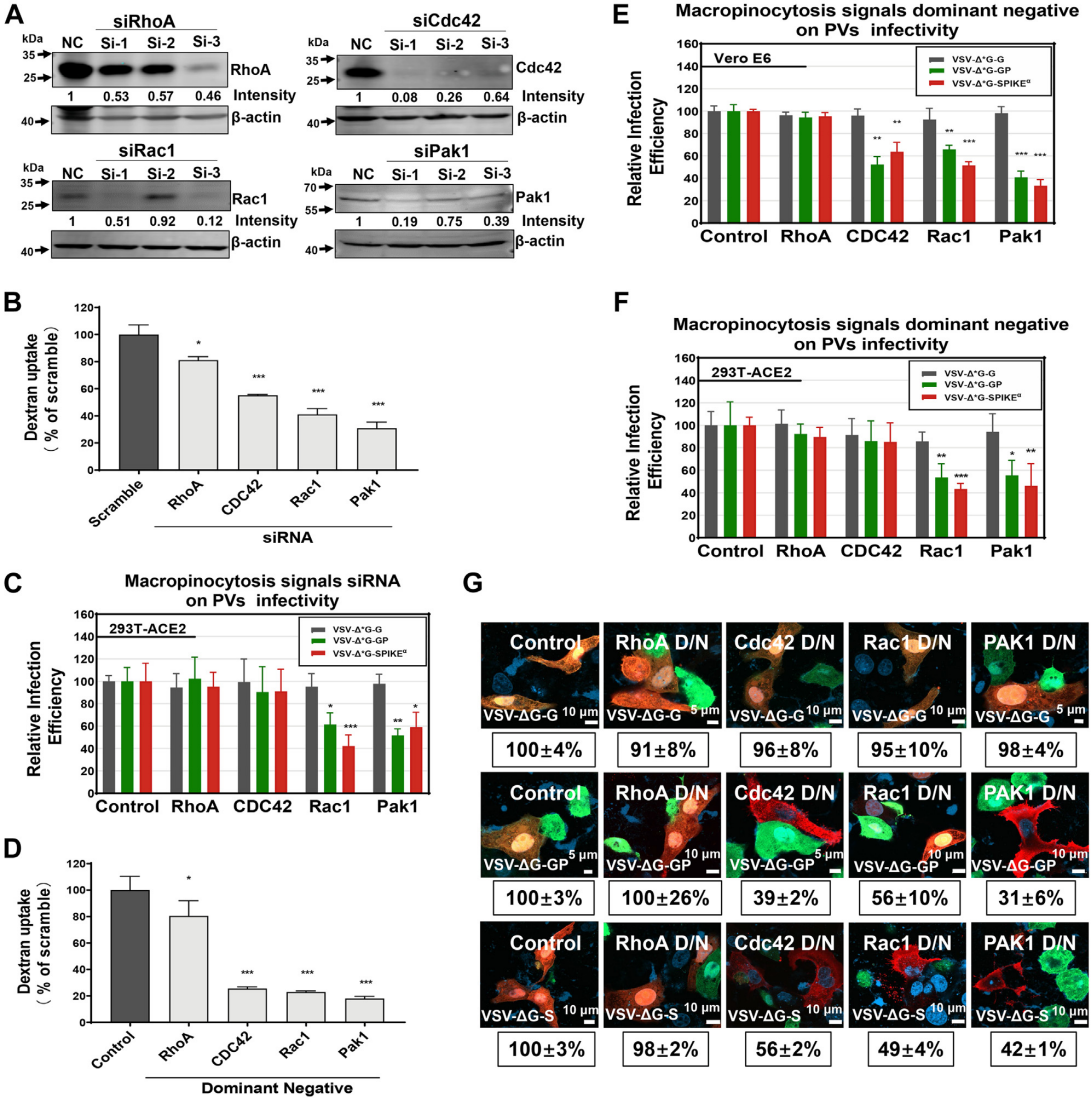
**Impairment of macropinocytosis signaling blocks SARS-CoV-2 entry.***A*, knockdown of RhoA, Cdc42, Rac1, and Pak1. HEK293 cells were transfected with siRNA targeting RhoA, Cdc42, Rac1, and Pak1. The expression of the indicated proteins was evaluated by Western blotting. The relative band intensity was determined by ImageJ, and the intensity of the control was set as 1. *B*, knockdown of RhoA, Cdc42, Rac1, and Pak1 by siRNA inhibited 70 kDa dextran uptake, which was determined by using flow cytometry. Each experiment was performed in triplicate, and the results are presented as the mean ± SD. *C*, knockdown of Rac1/Pak1 by siRNA blocks SARS-CoV-2 entry into HEK293T–ACE2 cells. HEK293T–ACE2 cells were transfected with siRNA targeting Pak1, RhoA, Cdc42, Rac1, or Pak1 or with scramble siRNA. Cells were then infected with the indicated VSV-ΔG-Luc pseudovirus at 48 h post-transfection. Luciferase values were normalized to scramble siRNA-transfected control cells. *D*, Vero E6 cells transfected with Cdc42-T17N, Rac1-T17N, Pak1-R299, or RhoA-T19N were incubated with 70 kDa dextran. The uptake of 70 kDa dextran was analyzed by using confocal laser scanning microscopy. *E*, Vero E6 cells and (*F*) HEK293T–ACE2 cells transfected with Cdc42-T17N, Rac1-T17N, Pak1-R299, or RhoA-T19N (as a control) were infected with VSV-ΔG-Luc pseudovirus. Luciferase values are normalized to the control. *G*, quantitative analysis of VSV-ΔG-EGFP pseudovirus infection in dominant negative–expressing Vero E6 cells. The dominant-negative form of the indicated proteins was labeled by an antimyc antibody in Vero E6 cells. VSV-ΔG-EGFP pseudovirus was incubated for 2 h at 37 °C, and the infectivity of VSV-ΔG-EGFP pseudovirus was analyzed by microscopy image analysis using confocal laser scanning microscopy. The scale bars represent 10 μm. ACE2, angiotensin-converting enzyme 2; EGFP, enhanced GFP; HEK293, human embryonic kidney 293 cell line; SARS-CoV-2, severe acute respiratory syndrome coronavirus 2; VSV, vesicular stomatitis virus.

The initiation of macropinocytosis requires the activation of a signaling cascade mediated by small GTPases and kinases. Rac1, Cdc42, and Pak1 are critical for MHV-induced macropinocytosis (18). Thus, we selected Rac1, Cdc42, and Pak1 to investigate whether SARS-CoV-2 entry was associated with the classical macropinocytosis signaling pathway. First, we evaluated whether endogenous Rac1, Cdc42, and Pak1 could influence SARS-CoV-2 entry. We knocked down the expression of Rac1, Cdc42, and Pak1 by siRNA, and RhoA was used as a control. The results demonstrated that RNAi significantly decreased the expression of the indicated proteins, as detected by Western blotting (Fig. 5*A*). The depletion of Rac1, Cdc42, and Pak1 significantly decreased the uptake of dextran, indicating that micropinocytosis was successfully inhibited by RNAi (Fig. 5*B*). Depletion of Rac1 and Pak1 significantly decreased SARS-CoV-2 entry, whereas a scrambled siRNA, along with a siRNA targeting RhoA or Cdc42, did not affect virus entry (Fig. 5*C*). These results demonstrated that SARS-CoV-2 induced macropinocytosis signals through Rac1 and Pak1 rather than through Cdc42, which is distinct from other coronaviruses, including MHV (18). We further confirmed this by the dominant-negative phenotype of the corresponding Rho GTPases. Vero E6 cells were transfected with Cdc42-T17N, Rac1-T17N, Pak1-R299, or RhoA-T19N (as a control), and the uptake of dextran was significantly decreased after Cdc42-T17N, Rac1-T17N, and Pak1-R299 transfection (Fig. 5*D*). Interestingly, we found that SARS-CoV-2 entry into Vero E6 cells was profoundly dependent on Rac1, Pak1, and CDC42 (Fig. 5*E*); however, in HEK293T–ACE2 cells, pseudovirus entry depended on Rac1 and Pak1 rather than on CDC42 (Fig. 5*F*). Furthermore, by using a pseudovirus system harboring the enhanced GFP (EGFP) reporter, we confirmed that Cdc42-T17N-, Rac1-T17N-, and Pak1-R299-transfected cells were resistant to EBOV-GP and SARS-CoV-2-spike pseudovirus entry but not VSV-G pseudovirus entry (Fig. 5*G*). Taken together, the results indicated that the small GTPase Rac1 and Pak1 kinases were involved in the SARS-CoV-2 entry process.

### Macropinocytosis was associated with SARS-CoV-2 spike–mediated cell-to-cell fusion

**Figure 6:**
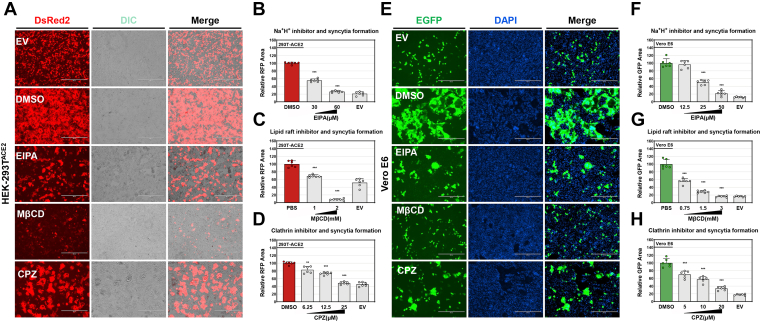
**An endocytosis inhibitor significantly decreases syncytia formation induced by the SARS-CoV-2 spike protein.***A*, HEK293T–ACE2 cells were transfected with S and DsRed2 plasmids. At 6 hpt, the cells were treated with different concentrations of EIPA, MβCD, CPZ, or DMSO as the control for another 18 h. Subsequently, images of syncytia were captured. The scale bar represents 400 μm. Quantification of the effects of (*B*) EIPA, (*C*) MβCD, and (*D*) CPZ on syncytia formation in HEK293T–ACE2 cells. *E*, Vero E6 cells were transfected with S and EGFP plasmids at 8 hpt, and the cells were treated with different concentrations of EIPA, MβCD, CPZ, or DMSO as the control for another 30 h. The cell nuclei were stained with DAPI. The scale bar represents 400 μm. Quantification of the effects of (*F*) EIPA, (*G*) MβCD, and (*H*) CPZ on syncytia formation in Vero E6 cells. The results are the mean ± SD from five fields per condition. ACE2, angiotensin-converting enzyme 2; CPZ, chlorpromazine hydrochloride; DAPI, 4′,6-diamidino-2-phenylindole; DMSO, dimethyl sulfoxide; EGFP, enhanced GFP; EIPA, 5-(*N*-ethyl-*N*-isopropyl) amiloride; HEK293T, human embryonic kidney 293T cell line; hpt, hours post-transfection; MβCD, methyl-β-cyclodextrin; SARS-CoV-2, severe acute respiratory syndrome coronavirus 2.

To investigate whether macropinocytosis was associated with viral spike–mediated cell-to-cell fusion, HEK293T–ACE2 cells were mock cotransfected or cotransfected with a plasmid expressing SARS-CoV-2 spike and a red fluorescent protein reporter plasmid as previously reported (27), followed by treatment with EIPA, CPZ, and MβCD at 6 h post-transfection (hpt). We observed that these inhibitors significantly decreased the number of nuclei in a syncytium and reduced syncytium size (Fig. 6, *A*–*D*). We further confirmed this phenomenon in Vero E6 cells, as shown in Figure 6, *E*–*H*. These data suggested that SARS-CoV-2 utilized macropinocytosis, clathrin-mediated endocytosis, and cholesterol-rich lipid rafts to initiate cell-to-cell fusion.

**Figure 7:**
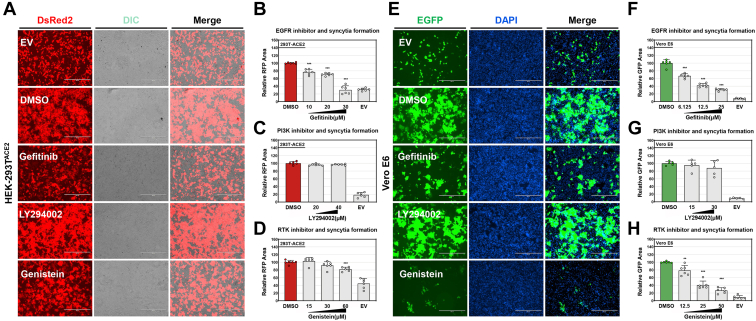
**A macropinocytosis inhibitor significantly decreases syncytia formation induced by SARS-CoV-2 spike protein.***A*, HEK293T–ACE2 cells were transfected with S and DsRed2 plasmids at 6 hpt. The cells were treated with the indicated concentration of gefitinib, LY294002, genistein, or DMSO as the control for another 18 h. Subsequently, images of syncytia were captured. The scale bar represents 400 μm. Quantification of the effects of (*B*) gefitinib, (*C*) LY294002, and (*D*) genistein on syncytia formation in HEK293T–ACE2 cells. *E*, Vero E6 cells were transfected with S and EGFP plasmids at 8 hpt. We perform this experiment and Figure 6 at same time, and we reuse empty vector (EV) image in Figure 6*E* here. The cells were treated with different concentrations of gefitinib, LY294002, genistein, or DMSO as a control for another 30 h. The cell nuclei were stained with DAPI. The scale bar represents 400 μm. Quantification of the effects of (*F*) gefitinib, (*G*) LY294002, and (*H*) genistein on syncytia formation in Vero E6 cells. The results are the mean ± SD from five fields per condition. ACE2, angiotensin-converting enzyme 2; DAPI, 4′,6-diamidino-2-phenylindole; DMSO, dimethyl sulfoxide; EGFP, enhanced GFP; HEK293T, human embryonic kidney 293T cell line; hpt, hours post-transfection; SARS-CoV-2, severe acute respiratory syndrome coronavirus 2.

For macropinocytosis, we next tested whether spike-induced cell-to-cell fusion required EGFR, PI3K, or other general tyrosine kinases. We found that gefitinib substantially reduced SARS-CoV-2 spike–mediated syncytium size in HEK293T–ACE2 and Vero E6 cells (Fig. 7, *A* and *B*). However, LY294002 failed to block SARS-CoV-2 spike–mediated cell-to-cell fusion and syncytium formation in HEK293T–ACE2 cells (Fig. 7, *A* and *C*) and possessed an inhibitory effect on SARS-CoV-2 entry. Syncytium formation was slightly blocked in Vero cells treated with LY294002 at a high concentration (Fig. 7, *E* and *G*). These results indicated that the PI3K pathway might not be important for SARS-CoV-2 spike–mediated cell-to-cell fusion and underscored that SARS-CoV-2 entry and viral spike–mediated cell-to-cell fusion exhibited a distinct macropinocytosis dependency. Interestingly, a general tyrosine kinase inhibitor, genistein, exhibited a different effect on the cell-to-cell fusion of SARS-CoV-2 in different cell lines. In particular, we found that genistein showed lower inhibitory activity on cell-to-cell fusion in HEK293T–ACE2 cells than in Vero cells, indicating that the inhibitory activity of genistein is cell type dependent (Fig. 7, *D* and *H*). However, the critical role of macropinocytosis during spike-mediated cell-to-cell fusion is clear.

## Discussion

Understanding the life cycle of SARS-CoV-2 is very important for combating the pandemic that it has caused. Cell entry and viral spike–mediated cell-to-cell fusion are two critical steps for coronavirus and SARS-CoV-2 replication (5, 37, 38). In this study, we demonstrated that both steps required macropinocytosis. More importantly, our study illustrated that inhibitors of macropinocytosis, clathrin-mediated endocytosis, and cholesterol-rich lipid rafts significantly decreased pseudotyped virus entry and significantly decreased S protein-mediated cell-to-cell fusion of SARS-CoV-2.

It is interesting that some of the intracellular signaling inhibitors can inhibit spike-induced cell‒cell fusion, which occurs on the membrane. As shown in Figure 7, gefitinib, an EGFR inhibitor that specifically inhibits EGFR autophosphorylation and prevents EGFR activation, was the most effective inhibitor of spike-induced cell‒cell fusion. In a recent study, the TMEM16 family was demonstrated to inhibit spike-induced syncytia, and drugs inhibiting TMEM16 proteins could block SARS-CoV-2 spike–induced syncytia (22). It has been reported that gefitinib could block TMEM16A-activated EGFR on the plasma membrane, which may be the possible reason for gefitinib inhibiting spike-induced cell‒cell fusion (22).

A recent study demonstrated that macropinocytosis was not required for SARS-CoV-2 entry in the HEK293T–ACE2 cell line using a pseudotyped virus (20), in contrast to our results. Interestingly, the results of another study were consistent with our conclusion and found that EIPA decreased SARS-CoV-2 replication (21), but the step in the SARS-CoV-2 life cycle that was inhibited is unknown (21). Our study demonstrated that EIPA significantly inhibited both SARS-CoV-2 cell entry and cell-to-cell fusion. In a previous report, macropinocytosis was shown to play a role in cell-to-cell spread rather than entry in the context of MHV infection (18). Our results indicated that macropinocytosis influences SARS-CoV-2 entry as well. This discrepancy may be due to the S protein of SARS-CoV-2 being distinct from that of MHV, which requires further investigation.

This study demonstrated that TMPRSS2 could restore the inhibition of SARS-CoV-2 entry and cell-to-cell fusion mediated by macropinocytosis inhibitors. In addition, TMPRSS2 could activate SARS-CoV-2 entry at the cell surface, suggesting that the combination of macropinocytosis inhibitors and TMPRSS2 inhibitors may be an effective method to cure SARS-CoV-2 in the clinic (10).

Macropinocytosis must be initiated by activating certain stimuli, and this activation involves downstream signaling through GTPases and kinases, including Rac1, Cdc42, and Pak1. In a previous study, MHV-activated macropinocytosis was shown to depend on signaling through Cdc42, Rac1, and Pak1 (18). However, using a dominant-negative and RNAi assays, we confirmed that for SARS-CoV-2 entry, Rac1 and Pak1 rather than Cdc42 were involved in signaling transduction in HEK293–ACE2 cells. Interestingly, in Vero E6 cells, using a dominant-negative assay, we confirmed that SARS-CoV-2 entry was dependent on Cdc42, Rac1, and Pak1. This indicated that SARS-CoV-2 entry into different cell lines might activate different macropinocytosis signaling pathways. Furthermore, the conflicting data on macropinocytosis involvement in SARS-CoV-2 entry and cell‒cell fusion were interesting. We assumed that SARS-CoV-2 entry and viral spike–mediated cell‒cell fusion mechanisms rely on different signaling pathways for initiation.

Overall, our data illustrated the critical role of macropinocytosis in SARS-CoV-2 entry and viral spike–mediated cell-to-cell fusion. This work will help us to understand SARS-CoV-2 infection and pathogenesis.

## Experimental procedures

### Plasmids, cells, viruses, and reagents

The full-length codon-optimized spike gene and EBOV-GP gene were synthesized at Sangon Biotech. The synthesized spike (18 amino acids deleted at the C-terminal region to facilitate pseudovirus packaging) was then subcloned into the eukaryotic expression vector pCAGGS. The dominant-negative forms of Rac1 (Rac1-T17N), Pak1 (Pak1-R299), RhoA (RhoA-T19N), and Cdc42 (Cdc42-T17N) were synthesized at Sangon Biotech with a myc tag at the N terminus and subcloned into the pcDNA3.1(+) vector. The TMPRSS2 plasmid was kindly provided by Xiaona Wang of Northeast Agricultural University (39). Human ACE2 was cloned from pCAGGS-HA-hACE2 (38) and subsequently cloned into the pB513B vector to generate pB513B-ACE2 (System Biosciences). Then, HEK293T cells were cotransfected with 3 μg pB513B-ACE2 and 1 μg helper vector expressing PB transposase (System Biosciences). Culture media were replaced at 48 hpt with growth media containing 1 μg/ml puromycin (Gibco) and replaced every 2 days as we previously described (5). HEK293T cells and Vero cells were maintained in Dulbecco's modified Eagle's medium (Gibco) with 10% fetal bovine serum (HyClone). EIPA (catalog number: HY-101840), MβCD (catalog number: HY-101461), LY294002 (catalog number: HY-10108), gefitinib (catalog number: HY-50895), Cyto-B (catalog number: HY-16928), and genistein (catalog number: HY-14596) were purchased from MCE; CPZ (catalog number: S5749) was purchased from SELLECK.

The SARS-CoV-2 B.1.1.7 (UK) strain used in this study (GenBank: MZ344997, GISAID virus name: hCoV-19/Hong Kong/HKU-210318-001/2020, accession number: EPI_ISL_1273444) was isolated from respiratory tract specimens of COVID-19 patients in Hong Kong and stored at the Physical Containment Level 3 Laboratory, Department of Microbiology, the University of Hong Kong (40).

### Pseudovirus production

VSV-G pseudotyped with VSV-ΔG-Luc or VSV-ΔG-EGFP was produced as previously described (41). Briefly, HEK293T cells were transfected with pCAGGS-SARS-CoV-2-S-, VSV-G-, or EBOV-GP-encoding plasmids using a jetPRIME kit (Polyplus Transfection). At 24 hpt, the medium from the transfected cells was removed and infected with VSV-ΔG-G reporter virus. After adsorption for 2 h, the pseudotyped virus was harvested at 24 h postinfection (hpi). Pseudovirus stocks were aliquoted and stored at −80 °C for later use.

### Antiviral activity assay

Vero E6 cells were treated with the indicated inhibitors or DMSO as a control for 45 min at 37 °C prior to inoculation with SARS-CoV-2 at an MOI of 0.05. After incubation for 1 h at 37 °C in the presence of the inhibitor, the medium was removed and replaced with fresh medium. At 6 h, cell lysates were collected, and RNA was extracted. Viral copies were determined by qRT‒PCR. For Caco2 cells, the cells were pretreated with the indicated inhibitors before infection with SARS-CoV-2 at an MOI of 0.5. Viral copies from cell lysates or supernatant were collected. Detection of viral genomes was performed by qRT‒PCR. The supernatant and cell lysate from the infected cells were harvested at 48 hpi for qRT‒PCR analysis as previously described (42).

### Viral entry assay

Vero E6 cells, Caco2 cells, or HEK293T–ACE2 cells expressing TMPRSS2 cultured in 96-well plates were treated with EIPA, CPZ, Cyto-B, LY294002, gefitinib, genistein, MβCD, or DMSO as a control for 45 min at 37 °C prior to inoculation with the indicated pseudovirus. For MβCD, cells were pretreated with MβCD for 45 min with no drug present during virus binding and infection. After incubation for 1 h at 37 °C in the presence of the inhibitor, the medium was removed and replaced with fresh medium. The activity of firefly luciferase was measured using the Luciferase Assay System (Promega) for quantitative determination of firefly luciferase activity at 24 hpi. Experiments were performed in triplicate.

### Fluid phase uptake assays

#### Fluorescence-activated cell sorting

To evaluate the effect of siRNA treatment on fluid phase uptake, siRNA-transfected HEK293T–ACE2 cells at 48 hpt were serum starved, pulsed with 1 mg/ml 70 kDa tetramethylrhodamine (TMR)–dextran for 20 min, and then harvested by treatment with trypsin. To remove surface-bound dextran, the cells were washed twice with cold PBS and once with low pH buffer (0.1 M sodium acetate, 0.05 M NaCl, pH 5.5) for 10 min. Cells were resuspended in PBS for fluorescence-activated cell sorting analysis with a SONY-MA900 Flow Cell Sorter System. Assays were performed in triplicate, and the results are displayed as the percentage of the mean fluorescence. Error bars represent the standard deviation between experiments.

#### Microscopy

Vero cells that were pretreated with the indicated inhibitors or transiently expressed dominant-negative RhoA, Cdc42, Rac1, or Pak1 were pulsed for 20 min with the fluid-phase marker 70 kDa TMR–dextran (1 mg/ml; Thermo Fisher Scientific). Surface-bound dextran was removed with a low pH wash (0.1 M sodium acetate, 0.05 M NaCl, pH 5.5) prior to formaldehyde fixation. Internalized TMR–dextran was analyzed by confocal laser scanning microscopy.

### Fusion assay

Vero E6 cells or HEK293T–ACE2 cells were seeded in a 24-well plate. At approximately 90% confluence, cells were cotransfected with 0.3 μg plasmid encoding 315GFP (43) or DsRed2 (an indicator of syncytium) with 0.8 μg pCAGGS-SARS-CoV-2-S. The medium was replaced with medium containing different inhibitors at 6 hpt. At 24 hpt, for Vero E6, the cells were fixed and stained with 4′,6-diamidino-2-phenylindole (Sigma), and for HEK293T–ACE2 cells, the cells were observed and photographed under available conditions. Six random fields for each well were selected to quantify the syncytia induced by SARS-CoV-2 spike protein. ImageJ (US National Institutes of Health) was used to calculate and analyze the GFP area of the image. The GFP area of the DMSO group was set as 100%.

### Cell cytotoxicity assay

The cytotoxicity of chemical inhibitors was verified using the CCK-8. Briefly, HEK293T–ACE2 cells (Caco 2 or Vero E6 cells) were seeded into a 96-well plate and incubated at 37 °C with 5% CO_2_ for 12 h. Then, 100 μl of cell culture medium containing the indicated chemical inhibitors was added. After 24 h of treatment, 10 μl of CCK-8 solution was added and incubated at 37 °C for 2 to 4 h. Then, the absorbance at 450 nm was measured by an absorbance microplate reader (ELx808).

### Dominant-negative assay

The dominant-negative targets RhoA, Rac1, Pak1, and Cdc42 were synthesized by Sangon and then cloned into the pcDNA3.1 (+) vector. HEK29T cells seeded into 96-well plates were transfected with the indicated dominant-negative expression vector, and transfected cells were inoculated with the indicated VSV-ΔG-Luc pseudotyped virus at 30 hpi. Luciferase activity was determined at 48 hpi.

For the VSV-ΔG-EGFP pseudotyped virus infection assay, Vero E6 cells were transfected with dominant-negative RhoA, Cdc42, Rac1, or Pak1. At 24 hpt, the cells were incubated with the indicated VSV-ΔG-EGFP pseudotyped virus. After 24 h, the cells were fixed in 3.7% paraformaldehyde for 20 min and permeabilized with 0.3% Triton X-100 for 10 min. The cells were washed three times with PBS and blocked with 2% bovine serum albumin for 1 h. The cells were incubated with a mouse antimyc antibody (Proteintech; catalog number: 60003-2-Ig: 1:1000 dilution) for 2 h at 37 °C and then with an Alexa Fluor 568–conjugated goat antimouse immunoglobulin G secondary antibody (Invitrogen). The nuclei were visualized by staining with 4′,6-diamidino-2-phenylindole. For each infection/transfection group, the mean fluorescence intensity of GFP for VSV-GFP pseudotyped virus infection in 200 transfected cells per group was analyzed by ImageJ. Experiments were performed in triplicate.

### RNAi assay

All siRNA duplexes targeting RhoA, Rac1, Pak1, and Cdc42 were synthesized by Gene Pharma, as listed in Table S1. HEK293T–ACE2 cells were seeded into 96-well plates, and the indicated siRNAs were transfected with X-tremeGENE siRNA transfection reagent (Roche; catalog number: 04476093001). Forty-eight hours later, transfected cells were infected with the pseudotyped virus, and the entry efficacy was analyzed by measuring luciferase activity.

### Western blotting

The detailed protocols of the Western blotting were as we previously described (38). The antibodies, diluted in Western blotting buffer (PBS, 5% bovine serum albumin, and 0.05% Tween), were mouse anti–beta-actin (Sigma; catalog number: A1978; 1:10,000 dilution), mouse antihemagglutinin tag (Sigma; catalog number: H9658; 1:4000 dilution), anti-Rac1 mouse McAb (Proteintech; catalog number: 66122-1-Ig; 1:2000 dilution), anit-Pak1 rabbit PolyAb (Proteintech; catalog number: 21404-1-AP; 1:1500 dilution), anti-Cdc42 rabbit PolyAb (Proteintech; catalog number: 10155-1-AP; 1:2000 dilution), anti-RhoA mouse McAb (Proteintech; catalog number: 66733-1-Ig; 1:1000 dilution), antimouse secondary DyLight 800-labeled antibodies (catalog number: 5230-0415; 1:10,000 dilution), and anti-rabbit secondary DyLight 800-labeled antibodies (catalog number: 5230-04125; 1:5000 dilution).

## Data availability

All data pertinent to this work are contained within this article or available upon request. For requests, please contact Yan-Dong Tang (tangyandong2008@163.com).

## Supporting information

This article contains supporting information.

## Conflict of interest

The authors declare that they have no conflicts of interest with the contents of this article.
